# Drumstick (*Moringa oleifera*) Flower as an Antioxidant Dietary Fibre in Chicken Meat Nuggets

**DOI:** 10.3390/foods8080307

**Published:** 2019-08-01

**Authors:** Pratap Madane, Arun K. Das, Mirian Pateiro, Pramod K. Nanda, Samiran Bandyopadhyay, Prasant Jagtap, Francisco J. Barba, Akshay Shewalkar, Banibrata Maity, Jose M. Lorenzo

**Affiliations:** 1 Division of Livestock Products Technology, ICAR-Indian Veterinary Research Institute, Bareilly 243122, India; 2 Eastern Regional Station, ICAR-Indian Veterinary Research Institute, Kolkata 700 037, India; 3 Centro Tecnológico de la Carne de Galicia, Adva. Galicia n° 4, Parque Tecnológico de Galicia, San Cibrao das Viñas, 32900 Ourense, Spain; 4 Poultry Processing Unit, Shalimar Hatcheries Limited, Grand Trunk Road, Golsi 713406, India; 5 Nutrition and Food Science Area, Preventive Medicine and Public Health, Food Science, Toxicology and Forensic Medicine Department, Universitat de València, Avda. Vicent Andrés Estellés, s/n, Burjassot, 46100 València, Spain

**Keywords:** lipid oxidation, natural antioxidant, sensorial properties, textural parameters, phenolic compounds, microbial analysis

## Abstract

The present work investigated the efficacy of Moringa flower (MF) extract to develop a functional chicken product. Three groups of cooked chicken nuggets—control (C), T1 (with 1% MF) and T2 (2% MF)—were elaborated and their physicochemical, nutritional, storage stability and sensory attributes were assessed during refrigerated storage at 4 °C up to 20 days. In addition, MF extracts were characterised in terms of chemical composition, total phenolic content and its components using high-performance liquid chromatography with a diode-array detector (HPLC-DAD), dietary fibre and antioxidant capacity. MF contained high protein (17.87 ± 0.28 dry matter), dietary fibre (36.14 ± 0.77 dry matter) and total phenolics (18.34 ± 1.16 to 19.49 ± 1.35 mg gallic acid equivalent (GAE)/g dry matter) content. The treated nuggets (T1 and T2) had significantly enhanced cooking yield, emulsion stability, ash, protein, total phenolics and dietary fibre compared to control. Incorporation of MF extract at 2% not only significantly reduced the redness/increased the lightness, but also decreased the hardness, gumminess and chewiness of the product compared to control. Moreover, the addition of MF extract significantly improved the oxidative stability and odour scores by reducing lipid oxidation during storage time. Sensory attributes of nuggets were not affected by the addition of MF extract and the products remained stable and acceptable even on 15th day of storage. These results showed that MF extract could be considered as an effective natural functional ingredient for quality improvement and reducing lipid oxidation in cooked chicken nuggets.

## 1. Introduction

Muscle foods are an excellent source of high-quality protein with high biological value. Moreover, the bioavailability of micronutrients such as iron, selenium, vitamins A, B12, folic acid, sodium, potassium and magnesium of these matrices is much higher than from plant sources [1]. In spite of being nutritious, meat is deficient in dietary fibre, a complex mixture of polysaccharides, which is naturally present as a part of plant material in cereals, vegetables, fruits and nuts. Lack of adequate quantity of dietary fibres in our diet has been involved in several health disorders such as colon cancer, obesity and cardiovascular diseases [2]. On the other hand, a fibre-rich diet is lower in energy density and richer in micronutrients thus reducing several disorders [3], and thereby promotes a healthier lifestyle. However, many food and food products, including meat products, not only lack of minimum amounts of dietary fibre [4] to fulfil the recommended dietary intake, but also differ in the quantity and composition of fibres.

As per the American Dietetic Association, the recommended dietary fibre intake for a healthy adult should be approximately 25 to 30 g/day, being the insoluble/soluble fibre ratio 3:1. Hence, to get the above health benefits of dietary fibre, attempts have been made to incorporate dietary fibres from various sources in meat product formulation to promote various technological benefits and the acceptability of meat products benefitting human health [5].

In a developing country like India, industrialisation, rapid urbanisation, globalisation as well as increasing number of women workforce are factors contributing to a rapidly growing inclination towards fast and convenient meat and food products [6]. However, meat and meat products contain an important amount of unsaturated fatty acids, which are sensitive to lipid oxidation [7,8]. Further, processing such as grinding, chopping, flaking, emulsification and cooking also accelerate lipid oxidation of meat and meat products [8]. Moreover, most meat products are processed using vegetable oils in order to overcome the problems of saturated fatty acids and cholesterol associated with animal fats [9,10]. These vegetable oils, with a high degree of polyunsaturation, accelerate oxidative processes, leading to meat quality deterioration. Therefore, lipid oxidation is considered the major causes of meat quality deterioration with development of undesirable flavours and odours, thus reducing the nutritional, sensorial and functional properties of meat products as well as consumer acceptability [11,12].

The use of antioxidants is considered as an effective method to minimise or inhibit lipid oxidation as well as inhibit the formation of toxic oxidation products in muscle foods, thereby improving the shelf-life of products [13,14,15]. Although synthetic antioxidants have been widely used in the meat industry, consumer concerns over safety issues of products have renewed the interest of food industry in search of antioxidants from natural sources [16]. Thus, both dietary fibre and natural antioxidants are considered as two important dietary fractions and could be very valuable in improving meat product quality and storage stability.

The natural ingredients having dual properties, i.e., a source of dietary fibre besides having antioxidant potential are known as antioxidant dietary fibres (ADF). ADF is defined as dietary fibre concentrate containing a significant content of natural antioxidants linked to the dietary fibre (DF) matrix in a single material [17]. To counter the shortcomings (low fibre content and oxidation) associated with meat and meat products, researchers and meat processors are continuously searching for natural ingredients and materials/extracts, especially of plant origin, to use as additives. The incorporation of ADF in meat products not only increases the shelf life during storage by inhibiting lipid oxidation due to presence of phenolic antioxidants, but also improve the texture, physicochemical and sensory properties of meat products [4,18,19]. 

*Moringa oleifera*, commonly known as horse radish tree or drumstick tree, is one of the most widely cultivated species native to the sub-Himalayan tracts of India. Almost all parts of this plant, like fruit (pods), gum, root, seed, bark, leaf, flowers and seed oil, are used as a nutritional and nutraceutical resources for human and animal diets [20]. The leaves and flowers are good source of protein and dietary fibre with an adequate profile of amino acids and ash [21,22]. The extracts of flowers of *M. concanensis* (fresh or dried) contain a great amount of ascorbic acid, polyphenols, tannins and flavonoids with high DPPH scavenging activity [23]. Considering the benefits of both dietary fibre and antioxidants in a single material, the objective of this study was to assess the potential use of Moringa flower (*M. oleifera*) as ADFs or functional ingredients in meat food system to enhance the nutritional quality, storage stability and acceptability of meat products.

## 2. Materials and Methods 

### 2.1. Reagents and Plant Materials 

Fresh mature moringa flowers (*M. oleifera*) were collected from a local market of Kolkata, India. Flowers were cleaned thoroughly to remove extraneous dirt, dried completely in a hot air oven at 45 ± 2 °C, ground in a grinder (Kenstar, Mumbai, India) and sieved (#60 mesh sieves). The powder obtained was stored in an air tight container at room temperature until further use. Chicken meat (breast) was obtained from West Bengal Livestock Development Corporation, Kolkata, India and kept under frozen storage at −18 °C till further processing. Different chemicals such as methanol, butylated hydroxytoluene (BHT), α-amylase, protease, amyloglucosidase trichloroacetic acid, sodium carbonate, 2-thiobarbituric acid, 2,2-diphenyl-1-picrylhydrazyl (DPPH), phosphate buffer, potassium ferricyanide, F-C reagents and gallic acid, caffeic acid, ferulic acid and quercertin were purchased from Sigma-Aldrich (Mumbari, India). All the chemicals used for this experiment were of analytical grade. 

### 2.2. Preparation of Moringa Flower Extract

Moringa flower (MF) was extracted using either aqueous (AE) or aqueous ethanol (AEH) as solvent (60:40, *v/v*). Briefly, for preparation of the extracts, 2 g of each MF was accurately weighed into separate conical flasks. To this, 100 mL of solvent were added and the whole content was held at room temperature (27 ± 1 °C) for 10 h, stirring frequently with a glass rod. The mixture was shaken at constant rate (500 rpm) using a shaker, vortexed at high speed for 10 min, and finally centrifuged (REMI NEYA 8, Kolkata, India) at 5000x rpm for 10 min. The content of each extract was then passed through Whatman filter paper No. 1 (HiMedia^®^, Mumbai, India). The resulting extract was kept in a container and stored at 2 °C for further studies. The extracts (aqueous or aqueous ethanol) obtained from different solvent were analysed for total phenolic contents (TPC), 1, 1 diphenyl-2-picrylhydrazil (DPPH) radicals scavenging activity and ferric reducing antioxidant power (FRAP) assays. The efficacy of the extracts was determined based on the weight of respective dry powders.

### 2.3. Analysis of Polyphenols and Antioxidant Capacity

The total phenolics content of the extracts was assessed using the Folin–Ciocalteu (F-C) method previously described by Singleton et al. [24], while in cooked nuggets the procedure described by Escarpa & González [25] with slight modifications was used. The results were expressed as mg gallic acid equivalents (GAE) /g of dry matter.

HPLC-DAD phenolic composition of MF extracts was carried out according the method of Zeb [26], using an Agilent 1260 Infinity HPLC system equipped with a quaternary pump, degasser, autosampler and diode-array (DAD) detector. An Agilent rapid resolution Zorbax Eclipse plus C18 (4.6 × 100 mm, 3.5 µm) column was used for the separation, using a gradient elution at 0.6 mL/min with gradient program (0–20 min, 95–75% A; 20–40 min, 75–50% A; 50–20%, 40–50 min A; 50–60 min, 20% A) with 1% formic acid in water as solvent A and methanol as solvent B. The chromatograms were obtained at 280 nm.

On the other hand, the method proposed by Fargere et al. [27] was used to determine the free radical scavenging activity (FRSA) using DPPH assay. Free radical scavenging activity (FRSA) was calculated using the following formula.

(1) $$FRSA\left(\%\right)=\frac{\left(absorbance\;control-absorbance\;sample\right)}{absorbance\;control}\times 100$$

The Ferric reducing antioxidant power (FRAP) assay was assessed following the procedure previously established by Oyaizu [28].

### 2.4. Determination of Dietary Fibre Content

The dietary fibre composition was analysed according to the enzymatic–gravimetric method [29]. A phosphate buffer was used to disperse the sample and a sequential enzymatic digestion using α-amylase, protease and amyloglucosidase was carried out. The total dietary fibre (TDF) was calculated as the sum of insoluble dietary fibre (IDF) and soluble dietary fibre (SDF) obtained from the enzymatic digestion.

### 2.5. Preparation of Chicken Nuggets 

Approximately 8 kg of meat sample (chicken breast meat, breast trimmings and chicken skin), obtained from West Bengal Livestock Development Corporation (West Bengal, India), was minced twice (minced through a 6 mm grinding plate followed by 4 mm plate) in a meat mincer. After mincing, meat samples were divided into three (3) different batches. The composition of ingredients for each batch was based on the standard formulation (Table 1). The first batch was considered as control (meat without any ADF), whereas MF at 1% and 2% level were used as ADF for chicken nuggets in formulations (T1 and T2) replacing equal per cent of breast trimmings.

All batches of minced meat samples were prepared separately in a bowl chopper. In order to prepare meat emulsion, salt, phosphate and nitrite were added and mixed to the meat using a bowl chopper. Ice flakes were added to keep temperature at ~8 ± 2 °C. Afterwards, condiments, dry spice mix, and fine wheat flour were added, and chopping was continued till uniform mixing of all the ingredients. The emulsion prepared was steam-cooked (100 °C) for 40 min to get cooked chicken meat nugget. The blocks were sliced uniformly to obtain small cubes (size) of chicken nuggets. The formulated nuggets (C, T1 and T2) were aerobically packaged in low density polyethylene (LDPE) pouches and kept under refrigerated conditions (4 ± 1 °C) to evaluate different physicochemical parameters, including storage stability (0, 5, 10, 15 and 20 days) and sensory attributes. The whole experiment was conducted three times and samples were analysed in duplicate.

### 2.6. Microbial Analysis 

Total plate count (cfu/g) was determined by using pour plate method according to Lorenzo et al. [30]. Ten grams of meat sample were homogenised in 90 mL of sterile peptone water (0.1%). Appropriate serial dilutions were prepared in 0.1% sterile peptone water and plated in duplicate with plate count agar, incubated at 37 °C for 48 h. Microbial colonies from the plates were counted and expressed as log cfu/g.

### 2.7. Physicochemical Analysis

Moisture, protein, fat and ash content of the cooked meat nugget samples were determined by the procedures previously described by Association of Official Analytical Chemists AOAC [31]. The pH of sample was determined by combination electrode digital pH meter (Benchtop pH meter, BR Biochem, PHS-25CW, New Delhi, India). Briefly, 10 g of sample was homogenised with the help of tissue homogeniser (Omni International, Kennesaw GA, United States) for approximately a minute in 50 mL of distilled water. The homogenised sample was kept for 5 minutes and then mixed again by shaking rod. The pH value was recorded by immersing the electrode directly into the suspension.

The colour profile of thick slice of chicken nugget or fresh chicken block was measured using Hunter Color Lab (Mini XE, Portable, HunterLab, Reston, VA, United States) to record Hunter L*, a*, and b* values. The instrument was calibrated using light trap/black glass and white tile provided with the instrument. The instrument was directly put on the surface of meat block and reading was taken at three different points. The textural properties of nuggets were evaluated using a texture analyser (Stable Micro System, Model TA. HDi, Surrey, UK). Texture profile analysis (TPA) was performed using central cores of five pieces of each sample (2 cm × 2 cm × 2 cm), which were compressed twice to 80% of the original height by a compression probe (P 75). A crosshead speed of 2 mm/s was used. The following parameters were determined; hardness (N/cm^2^), springiness (cm), cohesiveness, gumminess (N/cm^2^) and chewiness (N/cm).

Cooking yield of nuggets was determined by recording the weight of each meat block before and after cooking and expressed as percentage according to the following equation.

(2) $$Cooking\;yield\;\left(\%\right)=\frac{\left(wt\;ofcooked\;meat\;block\right)}{wt\;of\;raw\;meat\;block}\times 100$$

Expressible water was estimated using the method of Das et al. [32] with slight modifications. Approximately 5 g of cooked sample was weighed and placed on 2 layers of Whatman no. 1 filter paper. The sample was placed at the bottom of 50 mL centrifuge tube and centrifuged at 1500 g (Remi, Mumbai, India) for 15 min. Immediately after centrifugation, meat samples were reweighed and the percentage of expressible water was calculated according to the following equation.

(3) $$Expressible\;water\;\left(\%\right)=\frac{\left(initial\;weight-final\;weight\right)}{initial\;weight}\times 100$$

### 2.8. Thiobarbituric Acid Reacting Substances (TBARS) Values

The evaluation of lipid stability was performed by measuring TBARS values following the method proposed by Witte et al. [33]. The TBARS value was calculated as mg malonaldehyde (MDA) per kg of the sample by multiplying the absorbance value with a factor of 5.2.

### 2.9. Sensory Evaluation of Chicken Nuggets

Sensory evaluation of the control and treated chicken nuggets was used for various sensory attributes by trained and experienced panellists familiar with the characteristics of the meat product. Just before sensory evaluation, nugget samples (C, T1 and T2) were warmed in a microwave oven for 20 s, coded and then served to evaluate for general appearance, colour, flavour, binding, texture, juiciness and overall acceptability using 8-point descriptive scale [34], where 8 = extremely desirable and 1 = extremely undesirable. Plain potable water was provided to rinse the mouth in between the samples.

### 2.10. Statistical Analysis

The present study was repeated three times and, in each replication, measurements of all parameters were done in duplicate. The analysis was carried out using statistical package for the social sciences (SPSS) software (version 20.0, IBM SPSS, Armonk, NY, United States) and for storage study, data was analysed using two-way analysis of variance (ANOVA) with interaction using SPSS software where treatment (control, T1 and T2) and storage time (0, 5, 10, 15 and 20) were considered as factors. The experiment was carried out in 3 × 5 factorial design according to a completely randomised design. The obtained data were subjected to variance analysis, and Duncan’s multiple range test was used for comparing the means to find out the effect of antioxidant dietary fibres on various parameters and the differences were considered at α = 0.05 level. The values were presented as mean along with standard error (mean ± standard error).

## 3. Results and Discussion

### 3.1. Chemical Composition, Phenolic Content and Antioxidant Activity of MF Extract

The chemical composition and dietary fibre contents of the MF extract are shown in Table 2. The protein, ash and lipid content of MF were 17.87 ± 0.28%, 7.87 ± 0.45% and 2.95 ± 0.07%, respectively. In general, the chemical composition depends on the edible part of a plant being analysed. Our findings are in disagreement with the data reported by other authors in MF extract [35,36]. This fact could be attributed to the soil type, cultivars, stage of maturity of flowers and influence of the climatic or weather conditions in the region [37]. As presented in Table 2, MF extract had 36.14 ± 0.77% TDF, 3.90 ± 0.14% SDF and 32.24 ± 0.82% IDF contents. The content of total dietary fibre in MF extract is similar to that reported by Sánchez-Machado et al. [36]. The presence of great amount of IDF and SDF in the MF extract indicate that MF is a promising source of dietary fibre with a very good physiological effect; better than some cereals like wheat bran (2.9% SDF; 41.1% IDF), and oat bran (3.6% SDF; 20.2% IDF) as reported by Grigelmo-Miguel et al. [38].

In the present study, the aqueous ethanolic extract of MF presented the higher total phenolic compounds (TPC) (19.49 ± 1.35 mg GAE/g powder) followed by water extract (18.34 ± 1.16 mg GAE/g powder), although nonsignificant (Table 2). The higher TPC might be attributed to different degree of polarity of the solvents used for the extraction of polyphenolic compounds, and thus could have contributed significantly to the antioxidant and free radical scavenging activity. Similar observation was also demonstrated by Tekle et al. [39], who reported that the total polyphenolic content of MF extract was relatively higher in ethanolic extract compared to its water counterpart.

The phenolic compounds in MF extracts were identified and quantified as shown in Table 2. Among the phenolics, ferulic acid was present in high concentration and other compounds, including flavonoids (quercetin) were also present. In fact, antioxidant activities of plants mainly depend on the amount of phenolic acids (gallic acid, feluric acid and caffeic acid) and flavonoids (catechin, myricetin and quercetin). Several studies indicate that flavonoids are the main contributor for plant’s antioxidant activity [40,41]. However, different phenolics exhibit different antioxidant potential, and other compounds may influence the antioxidant activities [42]. The strong antioxidant activity exhibited by MF extracts could be due to abundance of ferulic acid and quercetin.

**Figure 1:**
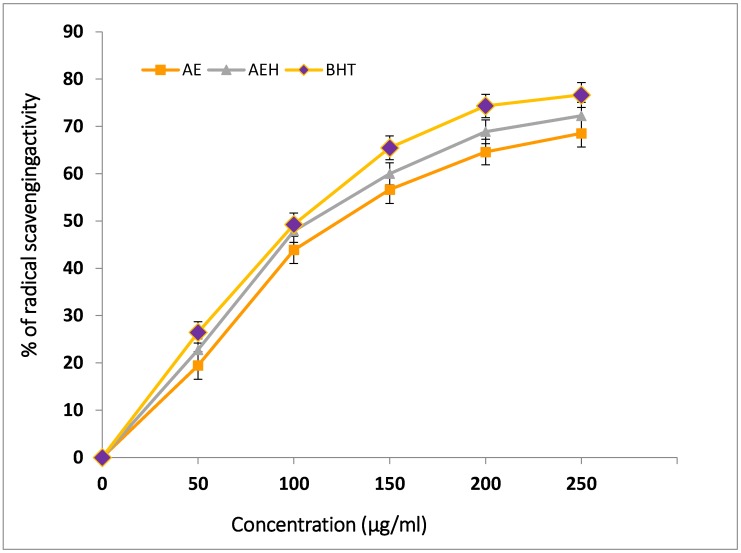
DPPH radical scavenging activity of MF extracts. AE = Aqueous extract; AEH = Aqueous ethanolic extract; BHT = Butylated hydroxytoluene.

According to DPPH scavenging activity, our results showed that aqueous ethanol extract of MF contained higher radical scavenging activity than aqueous extract (Figure 1). Both extracts demonstrated purple bleaching reaction at increased concentrations, showing the presence of compounds responsible as free radical scavengers thus reducing the initial DPPH concentration.

Figure 1 clearly illustrates that the radical scavenging activity of Moringa flowers in both extracts was comparable to butylated hydroxytoluene (BHT) at concentrations from 0 to 250 µg/mL. The levels of inhibition of DPPH radical by the AE, AEH and BHT were 68.52, 72.22 and 76.64%, respectively, and in a concentration-dependent manner. The BHT used as positive control displayed similar level of inhibition at 250 µg/mL, which was nonsignificantly different in comparison to AE. A high correlation exists between DPPH radical scavenging potential and TPC of plant extracts. A study conducted by Siddhuraju et al. [43] reported that at a dosage ranging from 0.2 to 0.6 mg of acetone extract, *M. oleifera* (pericarp of immature drumstick and flower) and *S. grandiflora* (flower and leaf) showed higher free radical scavenging activity (8.45–65.03%). Sreelatha and Padma [44] noticed that methanol extract of *M. oleifera* leaves significantly reduced DPPH radicals, though lower than our observed results. These variations could be due to difference in the polarity of solvents and geographical location of the plants [44].

In terms of IC_50_, the lowest value was shown by the positive control, BHT (118 µg/mL), followed by AEH (121.42 µg/mL) and AE (126.20 µg/mL) of MF extracts (Table 2). Alhakmani et al. [45] reported that DPPH radical scavenging activity of *M. oleifera* flower extract was compared with standard ascorbic acid. Although standard antioxidant had higher scavenging activity at all tested concentrations, the flower extract still showed good free radical scavenging activity. This radical scavenging activity of extracts might be related to the nature of phenolics, thus contributing to their electron transfer/hydrogen donating ability [22,43].

**Figure 2:**
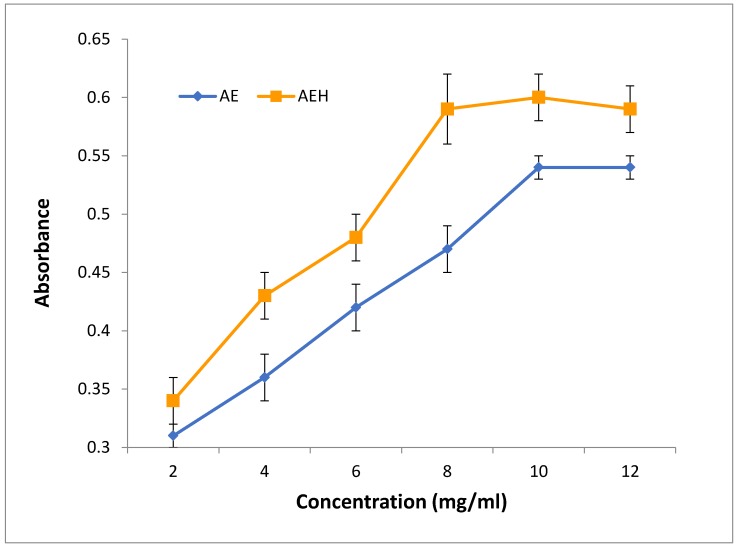
Ferric reducing antioxidant power of MF extracts. AE = Aqueous extract; AEH = Aqueous ethanolic extract.

Regarding FRAP assay, MF extract presented good activity on a dose dependent manner. As the aqueous ethanol-extracted MF extract exhibited an overall higher activity at the highest concentrations compared to water extract MF (Figure 2). This outcome showed that it was more efficient in extracting antioxidants from plant materials. In this regard, Tekle et al. [46] also found that ethanolic extract of M. oleifera leaf and flower exhibiting higher ferric reducing antioxidant activity (*p* < 0.05) compared to the synthetic antioxidant, BHT.

### 3.2. Effect of MF Extract on Physico-Chemical Properties of Meat Nuggets

Table 3 shows the influence of the MF extract on the physicochemical properties of meat nuggets. There was a significant (*p* < 0.05) decrease in emulsion pH with incorporation of MF extract at both levels used in this study. The highest pH value was observed in control (6.33) followed by T1 (6.25) and T2 (6.22) batches, although nonsignificant (*p* > 0.05). The decrease in pH of meat emulsion might be due to acidic pH value (5.44) of MF extract. Our findings agree with the results of Devatkal et al. [47], who noticed that kinnow rind extract significantly decreased the pH values of cooked goat meat patties due to its acidic pH. In a similar study, Habib et al. [48] also found a decrease in pH on the incorporation of pomegranate rind powder at three different levels. However, Das et al. [49] noticed that the use of *M. oleifera* leaves extract did not modify the pH of raw and cooked goat meat patties, whereas Hazra et al. [50] observed that the addition of *M. oleifera* leaves extract increased (*p* < 0.05) the pH values of cooked ground buffalo meat.

Statistical analysis showed that the moisture and lipid content did not differ significantly (*p* < 0.05) between control and treated samples (Table 3). Our findings agree with the data reported by Al-Juhaimi et al. [51], who observed that the moisture content of raw patties decreased as the percentage of *Moringa oleiferi* seed flour increased, but the decline rate was found to be not significant. In addition, Hazra et al. [50] also found that the inclusion of *Moringa oleiferi* leaf extract did not modify the moisture and lipid content of cooked ground buffalo meat. The protein content of the control nugget was 14.38%, whereas the treated nuggets (T1 and T2 groups) presented protein values of 15.27% and 16.32%, respectively. In fact, Moringa flowers have a very high crude protein content varying between 18.92 and 26.16% [35,36]. Therefore, the increase in protein percentage of chicken nuggets might be due to higher protein (17.87%) percentage of MF extract as found in this study. There was a significant difference in the percentage of ash content between control and treated nuggets. The increased ash content in treated chicken nuggets might be due to high ash content of MF (7.87%). A similar trend was reported by Al-Juhaimi et al. [51] who noticed that the ash content of raw patties increased as the percentage of *Moringa oleifera* seed flour increased in cooked ground buffalo meat.

The incorporation of MF significantly (*p* < 0.05) enhanced the emulsion stability of treated nuggets than the control (Table 3). The control and treated chicken nuggets (T1 and T2 groups) showed a 94.45%, 95.56% and 96.47% emulsion stability, respectively. The probable reason for increased emulsion stability could be due to the presence of dietary fibre in MF (36.14%). In this regard, Das et al. [18] also observed that bael pulp residue at 0.5% significantly improved (*p* < 0.05) the emulsion stability in goat meat nuggets. Similarly, Sarıçoban et al. [52] studied the effect of different concentrations (2.5%, 5%, 7.5% and 10%) of lemon albedo (raw and dehydrated) on the functional properties of emulsions and found an increase in the emulsion capacity at 5 percent of added emulsions. A similar trend was also observed in emulsion stability values. Malav et al. [53] reported that there was an increasing trend in emulsion stability with increase in levels of red kidney bean powder. The emulsion stability and cooking yield increased on incorporation of dietary fibre extracted from rice bran at different levels in meat batters [54].

On the other hand, a significant (*p* < 0.05) improvement in cooking yield was observed due to the incorporation of MF at different levels (Table 3). The higher percentage of cooking yield (97.83% and 97.26%) was observed in nuggets with MF than the control (96.79%). The articles available in the literature regarding this aspect indicate that non-meat ingredients with high dietary fibre content, when used in emulsion type of meat products, improve the cooking yield. A similar trend was reported by Ham et al. [55] who noticed that increasing lotus rhizome powder levels as a source of ADF lowered the cooking loss of emulsion sausages (5.89–6.25%) significantly (*p* < 0.05) than that of control sausage (7.31%). In addition, Anderson and Berry [56] also observed that 10% fat beef patties extended with pea fibre had high cooking yield. The probable reasons for the increased cooking yield of chicken nuggets with MF extract could mainly be attributed to the presence of high amount of dietary fibre and its ability to bind more water and fat as reported by Verma and Banerjee [4]. However, this finding disagrees with the data reported by Das et al. [49] who observed that the use of *M. oleifera* leaves extract did not influence the cooking loss of goat meat patties.

On the contrary, Hazra et al. [50] found that the inclusion of *M. oleifera* leaves extract showed a significant reduction in cooking loss of cooked ground buffalo meat. In this study, the expressible water content of nuggets ranged between 21.71% and 27.14% (Table 3). Although chicken nuggets with MF extract had lower expressible water, indicating higher water-holding capacity (WHC), there was no significant effect (*p* > 0.05) compared to control group. This result agrees with those reported by Vural et al. [57] who observed that the use of sugar beet fibre increased the water-holding capacity of frankfurter without any significant changes on sensory properties. In addition, Chang and Carpenter [58] also reported that more water was retained in frankfurters with an increase in oat bran level. Our findings clearly indicate that fibre can be used in cooked meat products to increase the WHC.

Addition of MF significantly (*p* < 0.001) increased the total dietary fibre (TDF) and total phenolics content (TPC) in chicken nuggets (Table 3). The TDF content was the highest in nuggets with 2% MF extract (2.03%) followed by T1 group (1.39%), while the lowest values were found for control samples (0.76%). This outcome agrees with the data reported by Verna et al. [6] who noticed that the incorporation of guava powder as ADF significantly improved the TDF content of meat nuggets. Similarly, TPC content was significantly higher in treated nuggets (0.789 and 1.121 mg GAE/g for T1 and T2 groups, respectively) than control samples (0.059 mg GAE/g). Such a high dietary fibre and TPC level in treated chicken nuggets might be due to use of MF extract, which had very high phenolic content (36.14 mg/g dry powder) and good source of dietary fibre (36.14%). This result agrees with the data observed by Das et al. [49] who showed that TPC of cooked goat meat patties with *M. oleifera* leaves extract was significantly (*p* < 0.05) higher compared to control group.

In addition, Das et al. [18] also found significantly increased (*p* < 0.05) TPC by incorporating bael pulp residue as ADF in meat products. Moreover, Das et al. [34] reported that sheep meat nuggets incorporated with 1% and 1.5% litchi fruit pericarp extract had significantly higher TPC than control nugget.

The incorporation of MF extract did not influence (*p* > 0.05) the textural parameters (hardness, cohesiveness, gumminess and chewiness) of the product, although slightly lower hardness values were found in T2 group, indicating softer chicken nuggets compared to control (Table 3). A similar trend was found by Verma et al. [6] who observed that hardness, adhesiveness, cohesiveness, gumminess and chewiness values were not significantly affected (*p* > 0.05) by the addition of guava powder. Similarly, Choi et al. [54] studied the addition of rice bran fibre on the textural properties of heat-induced gel, and found that not only hardness, but springiness, cohesiveness, gumminess and chewiness were also lower in samples with added rice bran fibre relative compared to control treatment. Moreover, Ham et al. [55] reported that textural properties, notably hardness, cohesiveness, and gumminess of cooked sausages were unaffected (*p* > 0.05) even when formulated with different levels of lotus rhizome powder. Other textural properties recorded in this study were nonsignificantly different (*p* > 0.05) between control and treatment chicken nuggets, though all the values decreased with increasing levels of MF. Wan Rosli et al. [39] while studying the textural properties of chicken patties formulated with different levels (0, 25% or 50%) of grey oyster mushroom, as a source of fibre and fat replacer, found lower cohesiveness, gumminess and chewiness values compared to control.

### 3.3. Effect of MF on pH, TBARS Values and Total Plate Count of Chicken Nuggets during the Storage Time

The pH values of the control and treated chicken nuggets, which were aerobically packaged and stored under refrigerated conditions, were evaluated at five-day intervals up to 20th day and are presented in Table 4. Statistical analysis displayed significant difference (*p* < 0.05) between storage days and treatments. As storage period progressed, a significant (*p* < 0.05) increase in pH values was observed in both the control and treated nuggets. The pH values increased from 6.30 ± 0.02 to 6.50 ± 0.01, from 6.27 ± 0.01 to 6.36 ± 0.01 and from 6.26 ± 0.01to 6.37 ± 0.01 from day 0 to day 20 for control, T1 and T2, respectively. The increase in pH during the storage period of meat product might be because of accumulation of metabolites due to the growth of Gram-negative bacteria like Pseudomonas, Moraxella, Acinetobacter, etc. [59]. Das et al. [60] also observed an increase in pH of ground and cooked meat added with curry leaf (*Murraya koenigii*) during the storage time. However, the increase in pH during the refrigerated period was significantly (*p* < 0.05) less in treated samples compared to control treatment, which might be due to the inhibitory effects of MF extract on oxidation of protein and lipid and some antimicrobial effects of the plant powder [61].

The mean ± SE values of total plate count of aerobically packaged control and treated chicken nuggets (T1 and T2) during refrigerated storage up to 20 days is presented in Table 4. During storage, a significant increase (*p* < 0.05) in microbial count was observed in control and treated products (T1 and T2) at each interval of storage period except on 0 day, where the counts were comparable. Total plate count increased from 2.74 ± 0.06 to 6.46 ± 0.04 log_10_ cfu/g, from 2.64 ± 0.09 to 4.66 ± 0.10 log_10_ cfu/g and from 2.71 ± 0.04 to 4.51 ± 0.05 log_10_ cfu/g, from day 0 to day 20 for control, T1 and T2 groups, respectively. An increase of total plate count of chicken sausage during refrigerated storage was noticed by Sallam et al. [62]. However, at the end of storage time, the total plate count of treated chicken nuggets was significantly lower compared to the control group (6.46 vs. 4.66 and 4.51; *p* < 0.001 for control, T1 and T2 groups, respectively). This result might be due to its richness in polyphenolic compounds [45] exerting antimicrobial [61] effects.

It has been well documented by some researchers that polyphenols from MF extract have microbial activities against a number of pathogenic bacteria [61]. The effectiveness of MF extract in lowering the total plate count of chicken nuggets are in agreement with the previous findings reported by Das et al. [18] who observed that incorporation of bael pulp residue as ADF was very much effective (*p* < 0.05) in controlling the microbial counts in goat meat nuggets throughout the 20 days of storage period.

The TBARS values of aerobically packaged chicken nuggets studied at regular intervals up to 20 days under refrigerated storage conditions is presented in Table 4. The TBARS values of chicken nuggets, irrespective of treatments, increased significantly (*p* < 0.05) from 0.37 ± 0.01 to 1.94 ± 0.05 mg MDA/kg, from 0.36 ± 0.01 to 0.84 ± 0.02 mg MDA/kg and from 0.36 ± 0.01 to 0.81 ± 0.01 mg MDA/kg, from day 0 to day 20 for control, T1 and T2 treatments, respectively. This increase in TBARS value throughout the storage period might be due to the lipid oxidation and production of volatile metabolites in presence of oxygen during aerobic storage [63,64,65,66]. Although TBARS value of all treated groups increased, but the rate of increase was comparatively slower in case of treated nuggets indicating more oxidative stability due to the presence ADF from MF extract. As observed on day 15, treated nuggets were within the spoilage limit, whereas during the same period, control nuggets (1.38) crossed the acceptable limit of 1 mg MDA/kg. The polyphenolic compounds present in the MF extract may be the reason for the strong antioxidant ability as reported by different researchers [22,47].

While comparing the treated groups, it was found that chicken nuggets in T2 group retarded the oxidation process more efficiently by maintaining TBARS values below the unacceptable range during 20 days storage. A similar result was found by Das et al. [50], who showed that TBARS values of cooked goat meat patties with *M. oleifera* leaves extract was 47% less than control group. Sáyago-Ayerdi et al. [67] also reported similar findings using grape antioxidant dietary fibre in chicken hamburgers. In addition, this outcome agrees with those reported by other authors [9,10,68,69,70,71] who observed that the addition of natural antioxidant decrease the TBARS values of meat products.

### 3.4. Effect of MF on Instrumental Colour Stability during the Storage Time

The incorporation of MF extract significantly changed the colour value of treated chicken nuggets compared to control during storage time (Table 5). A significant increase in the lightness values, in treated chicken nugget (T1 and T2 groups) was observed on 0 day in comparison to control. However, the reduction in lightness value of treated chicken nuggets was noticed after 10 days of storage period which may be attributed to the dilution of meat pigment in other meat products due to the presence of non-meat ingredients.

### 3.5. Effect of MF on Sensory Attributes of Chicken Nuggets during the Storage Time

Although both the control and treated chicken nuggets were comparable for all the above sensory attributes up to the 5th day of storage, the control nuggets received lower sensory scores thereafter (Table 6). This finding agrees with data reported by Das et al. [49] who showed that the addition of *M. oleifera* leaves extract had no effect on the sensory attributes. In addition, Muthukumar et al. [72] noticed that the of *M. oleifera* leaves extract did not modify colour, odour, flavour or texture, and all the products were equally acceptable as evidenced by the overall acceptability scores. The sensory attributes for treated nuggets (T1 and T2 groups), even though decreased with increasing storage time, were acceptable up to the 15th day of storage. On the other hand, the sensory scores for appearance of control, T1 and T2 treatments decreased from 6.77 ± 0.15 to 5.62 ± 0.19, from 7.02 ± 0.010 to 6.44 ± 0.10 and from 6.72 ± 0.15 to 6.38 ± 0.10, respectively. There was a significant decrease (*p* < 0.05) in appearance attribute of treated nuggets from day 15 onwards; whereas, in control samples, the values were even lower on 10th day of storage time.

This fact may be due to protective effect of MF extract preventing the fading of colour of the chicken nuggets. Control group received significantly lower flavour score on day 10 and development of rancid odour was noticed on 15th day of storage time, which might be the influencing factor to reduce the flavour and acceptability scores and was, therefore, rejected by the panellists. On the other hand, MF extract containing ADF which might have acted as stabilising agent for retaining the flavour by inhibiting lipid oxidation in treated chicken nuggets. There was hardly any remarkable variation, and the texture of nuggets with MF was comparable to control (*p* > 0.05) up to 10th day. Sensory texture was not done in case of control from 15th day onwards, as rancid odour was detected. Likewise, a significant decrease (*p* < 0.05) in juiciness score was observed in both control and treated (T1 and T2) products from 10th day onwards. However, chicken nuggets containing MF extract were found to be more juicer than the control group, which could be attributed to the increased moisture retention of the product during cooking.

As far as overall acceptability is concerned, the products also followed the same pattern that was observed for other sensory attributes. The control sample received a lower overall acceptability score, although nonsignificant (*p* < 0.05), on day 5 than treated samples, and was found to have rancid odour on day 15. On the other hand, overall acceptability scores obtained from T1 and T2 groups remained stable and were acceptable even on 15th day of storage. This could be due to incorporation of MF extract which might have extended the shelf-life. It is well documented by many researchers that meat products incorporated with natural antioxidants have higher flavour and overall acceptability scores during storage owing to the colour and flavour stabilising effect of them by inhibiting lipid and protein oxidation [73,74,75,76,77,78,79].

## 4. Conclusions

The results indicated that Moringa flower (MF) is a source of dietary fibre and also contains great antioxidant potential such as ferric reducing antioxidant power and radical scavenging activity. The addition of MF extract in chicken meat nuggets improved the cooking yield and dietary fibre content without affecting the acceptability of the meat product. Moreover, MF extract increased the lipid stability, odour score and shelf-life of chicken nuggets during 20 days of refrigeration storage. Therefore, MF extract could be used as a safe, natural and valuable antioxidant to the meat food industry, apart from offering its functional health promoting benefits.

## Figures and Tables

**Table 1:** Formulation of chicken nuggets with different levels of Moringa flower (MF) as antioxidant dietary fibre.

Ingredients (%)	Treatment		
	Control	T1	T2
Chicken meat	32.0	32.0	32.0
Breast trimming	20.0	19.0	18.0
Chicken skin	10.0	10.0	10.0
Ice flakes	20.0	20.0	20.0
Refined vegetable oil	8.00	8.00	8.00
Salt	1.50	1.50	1.50
Condiments *	4.00	4.00	4.00
Refined wheat flour	2.40	2.40	2.40
Dry spice mix **	1.80	1.80	1.80
Sodium nitrite (ppm)	150	150	150
Poly-phosphate	0.30	0.30	0.30
ADF powder %	0.0	1.0	2.0

Treatments: Control = no additive; T1 = 1.0% Moringa flower (MF) extract and T2 = 2.0% Moringa flower extract. * Condiments: garlic and onion (4:1). ADF = Antioxidant dietary fibre. ** Dry spice mix (18 g/kg nuggets)—aniseed, black pepper, capsicum, caraway seed, cardamom, cinnamon, cloves, coriander powder, cumin seed, turmeric and dried ginger.

**Table 2:** Proximate composition, total phenolic content (TPC), IC_50_ (µg/mL) and phenolic compounds of Moringa flower (mean values ± error standard of six samples).

**Proximate composition (g/100 g dry matter)**	
Protein	17.87 ± 0.28
Lipid	2.95 ± 0.07
Ash	7.87 ± 0.45
Total dietary fibre (TDF)	36.14 ± 0.77
Soluble dietary fibre (SDF)	3.90 ± 0.14
Insoluble dietary fibre (IDF)	32.24 ± 0.82
Nonstructural carbohydrates (NSC)	35.17 ± 1.25
**Antioxidant capacity**	
TPC (mg GAE/g dry matter) from AE	18.34 ± 1.16
TPC (mg GAE/g dry matter) from AEH	19.49 ± 1.35
IC_50_ µg/mL from aqueous extract	126.20 ± 1.45
IC_50_ µg/mL from aqueous ethanol extract	121.42 ± 1.28
Phenolic compounds (mg/kg dry matter)	
Caffeic acid	ND
Ferulic acid	270.08 ± 3.78
Quercertin	15.14 ± 0.40
Gallic acid	ND

AE = Aqueous extract; AEH = Aqueous ethanolic extract, ND = Not detected, IC = Inhibitory concentration.

**Table 3:** Effect of Moringa flower on physico-chemical parameters of chicken nuggets (mean values ± error standard of six samples).

Parameters	Treatments			Sig.
	Control	T1	T2	
Emulsion pH	6.33 ± 0.02 ^a^	6.25 ± 0.02 ^b^	6.22 ± 0.02 ^b^	**
Emulsion Stability (%)	94.45 ± 0.10 ^c^	95.56 ± 0.09 ^b^	96.47 ± 0.29 ^a^	**
Cooking yield (%)	96.79 ± 0.07 ^b^	97.26 ± 0.09 ^a^	97.83 ± 0.22 ^a^	*
Total phenolic content (mg GAE/g)	0.059 ± 0.02 ^c^	0.789 ± 0.09 ^b^	1.121 ± 0.15 ^a^	***
Expressible water (%)	27.14 ± 1.17	24.42 ± 3.00	21.71 ± 2.05	ns
**Chemical composition (g/100 g)**				
Moisture	67.29 ± 0.54	66.36 ± 0.82	65.74 ± 0.56	ns
Protein	14.38 ± 0.34 ^a^	15.27 ± 0.29 ^ab^	16.32 ± 0.66 ^b^	**
Fat	13.76 ± 0.49	14.06 ± 0.35	14.69 ± 0.62	ns
Ash	2.37 ± 0.49 ^a^	2.64 ± 0.13 ^ab^	2.91 ± 0.89 ^b^	*
Total dietary fibre	0.76 ± 0.03 ^a^	1.39 ± 0.04 ^b^	2.03 ± 0.06 ^c^	***
**Textural parameters**				
Hardness (N/cm^2^)	69.71 ± 2.43	65.52 ± 3.89	64.33 ± 6.49	ns
Springiness (cm)	0.67 ± 0.05	0.65 ± 0.02	0.63 ± 0.02	ns
Cohesiveness	0.30 ± 0.02	0.33 ± 0.01	0.34 ± 0.01	ns
Gumminess (N/cm^2^)	20.79 ± 0.02	20.01 ± 1.44	18.13 ± 3.04	ns
Chewiness (N/cm)	14.25 ± 2.25	13.79 ± 1.63	11.95 ± 2.54	ns

Treatments: Control = no additive; T1 = 1.0% Moringa flower extract and T2 = 2.0% Moringa flower extract. ^a–c^ Mean values in the same row not followed by a common letter differ significantly. Sig. Significance; ns: not significant; * *p* < 0.05; ** *p* < 0.01; *** *p* < 0.001.

**Table 4:** Effect of Moringa flower on pH, thiobarbituric acid reactive substances (TBARS) and microbial count (log cfu/g) of chicken nuggets during the storage time (mean values ± error standard of six samples).

Treatment	Storage Time (Days)					Sig.
	0	5	10	15	20	
**pH**						
Control	6.30 ± 0.02 ^d^	6.34 ± 0.03 ^cd^	6.39 ± 0.03 ^bcx^	6.45 ± 0.04 ^abx^	6.50 ± 0.01 ^ax^	***
T1	6.27 ± 0.01 ^c^	6.29 ± 0.01 ^bc^	6.32 ± 0.01 ^aby^	6.33 ± 0.01 ^aby^	6.36 ± 0.01 ^ay^	***
T2	6.26 ± 0.01 ^d^	6.30 ± 0.02 ^cd^	6.32 ± 0.01 ^bcy^	6.36 ± 0.01 ^aby^	6.37 ± 0.01 ^ay^	***
Sig.	ns	ns	***	***	***	
**TBARS (mg malonaldehyde/kg of sample)**						
Control	0.37 ± 0.01 ^e^	0.53 ± 0.01 ^dx^	0.89 ± 0.01 ^cx^	1.38 ± 0.02 ^bx^	1.94 ± 0.05 ^ax^	***
T1	0.36 ± 0.01 ^d^	0.38 ± 0.01 ^dy^	0.45 ± 0.01 ^cy^	0.58 ± 0.01 ^by^	0.84 ± 0.02 ^ay^	***
T2	0.36 ± 0.01 ^d^	0.37 ± 0.02 ^dy^	0.42 ± 0.01 ^cy^	0.52 ± 0.01 ^bz^	0.81 ± 0.01 ^ay^	***
Sig.	ns	***	***	***	***	
**Total Plate Count (log cfu/g)**						
Control	2.74 ± 0.06 ^e^	4.10 ± 0.07 ^dx^	5.13 ± 0.06 ^cx^	6.12 ± 0.08 ^bx^	6.46 ± 0.04 ^ax^	***
T1	2.64 ± 0.09 ^d^	3.37 ± 0.06 ^cy^	3.84 ± 0.07 ^by^	4.10 ± 0.05 ^by^	4.66 ± 0.10 ^ay^	***
T2	2.71 ± 0.04 ^d^	3.52 ± 0.12 ^cy^	3.83 ± 0.10 ^by^	4.02 ± 0.06 ^by^	4.51 ± 0.05 ^ay^	***
Sig.	ns	***	***	***	***	

Treatments: Control = no additive; T1 = 1.0% Moringa flower extract and T2 = 2.0% Moringa flower extract. ^a–e^ Mean values in the same row not followed by a common letter differ significantly among storage times. ^x–y^ Mean values in the same column not followed by a common letter differ significantly among treatments. Sig. Significance; ns: not significant; *** *p* < 0.001.

**Table 5:** Effect of Moringa flower on instrumental colour of chicken nuggets during the storage time (mean values ± error standard of six samples). Control = no additive; T1 = 1.0% Moringa flower extract; T2 = 2.0% Moringa flower extract.

Treatment	Storage Time (Days)					Sig.
	0	5	10	15	20	
L * (Lightness)						
Control	29.95 ± 1.20 ^ay^	26.21 ± 0.28 ^b^	25.49 ± 0.23 ^bcx^	23.89 ± 0.37 ^cdx^	22.52 ± 0.43 ^dx^	***
T1	33.80 ± 0.32 ^ax^	26.21 ± 0.28 ^b^	20.64 ± 0.44 ^cy^	19.22 ± 0.32 ^dy^	18.01 ± 0.26 ^ey^	***
T2	34.78 ± 0.44 ^ax^	26.21 ± 0.28 ^b^	19.97 ± 0.64 ^cy^	19.53 ± 0.59 ^cdy^	18.51 ± 0.26 ^dy^	***
Sig.	***	***	***	***	***	
a * (Redness)						
Control	13.77 ± 0.26 ^ax^	12.25 ± 0.20 ^bx^	6.73 ± 0.17 ^cy^	5.62 ± 0.15 ^cz^	5.11 ± 0.07 ^dy^	***
T1	12.78 ± 0.23 ^ax^	11.35 ± 0.15 ^az^	7.54 ± 0.12 ^bx^	6.58 ± 0.08 ^by^	5.44 ± 0.16 ^cy^	***
T2	11.01 ± 0.41 ^ay^	10.72 ± 0.13 ^by^	8.39 ± 0.08 ^cx^	7.85 ± 0.10 ^dx^	7.26 ± 0.16 ^dx^	***
Sig.	***	***	***	***	***	
b * (Yellowness)						
Control	13.11 ± 0.26 ^a^	11.81 ± 0.25 ^bxy^	7.98 ± 0.07 ^cx^	7.33 ± 0.13 ^dx^	6.92 ± 0.17 ^dx^	***
T1	13.70 ± 0.21 ^a^	11.41 ± 0.13 ^by^	6.30 ± 0.13 ^cy^	6.44 ± 0.16 ^cy^	5.68 ± 0.25 ^dy^	***
T2	13.99 ± 0.52 ^a^	12.30 ± 0.10 ^bx^	6.45 ± 0.29 ^by^	6.07 ± 0.20 ^by^	6.18 ± 0.09 ^by^	***
Sig.	***	***	***	***	***	

^a–e^ Mean values in the same row not followed by a common letter differ significantly among storage times. ^x–y^ Mean values in the same column not followed by a common letter differ significantly among treatments. Sig. Significance; ns: not significant; *** *p* < 0.001.

**Table 6:** Effect of Moringa flower on sensory attributes of chicken nuggets during the storage time (mean values ± error standard of six samples). Control = no additive; T1 = 1.0% Moringa flower extract; T2 = 2.0% Moringa flower extract.

Treatment	Storage Time (Days)					Sig.
	0	5	10	15	20	
**Appearance**						
Control	6.77 ± 0.15	6.75 ± 0.10 ^xy^	6.27 ± 0.27 ^aby^	6.19 ± 0.18 ^b^	5.62 ± 0.19 ^c^	**
T1	7.02 ± 0.10 ^a^	6.97 ± 0.01 ^ax^	6.94 ± 0.19 ^ax^	6.69 ± 0.08 ^ab^	6.44 ± 0.10 ^b^	**
T2	6.72 ± 0.15	6.61 ± 0.13 ^y^	6.66 ± 0.18 ^y^	6.58 ± 0.12	6.38 ± 0.10	**
Sig.	ns	*	**	**	ns	
**Flavour**						
Control	6.84 ± 0.18 ^a^	6.82 ± 0.09 ^a^	6.36 ± 0.47 ^b^	ND	ND	***
T1	6.97 ± 0.06 ^a^	6.93 ± 0.06 ^a^	6.72 ± 0.08 ^ab^	6.47 ± 0.17 ^bc^	6.33 ± 0.10 ^c^	***
T2	6.81 ± 0.09 ^a^	6.76 ± 0.09 ^a^	6.70 ± 0.16 ^a^	6.52 ± 0.11 ^ab^	6.27 ± 0.13 ^b^	***
Sig.	ns	ns	**	ns	ns	
**Texture**						
Control	6.72 ± 0.14	6.65 ± 0.09	6.52 ± 0.20	ND	ND	ns
T1	6.96 ± 0.10 ^a^	6.79 ± 0.12 ^ab^	6.50 ± 0.20 ^b^	6.42 ± 0.17 ^b^	6.38 ± 0.09 ^b^	***
T2	6.73 ± 0.11	6.78 ± 0.13	6.82 ± 0.11	6.66 ± 0.18	6.50 ± 0.13	ns
Sig.	ns	ns	ns	ns	ns	
**Juiciness**						
Control	6.72 ± 0.20 ^ab^	6.98 ± 0.12 ^a^	6.50 ± 0.15 ^b^	ND	ND	***
T1	6.80 ± 0.19	6.74 ± 0.22	6.47 ± 0.20	6.40 ± 0.12	6.30 ± 0.10	ns
T2	6.75 ± 0.10	6.75 ± 0.13	6.70 ± 0.19	6.52 ± 0.11	6.38 ± 0.10	ns
Sig.	ns	ns	ns	ns	ns	
**Overall acceptability**						
Control	6.80 ± 0.16 ^a^	6.76 ± 0.008 ^ay^	6.33 ± 0.27 ^by^	ND	ND	***
T1	7.02 ± 0.07 ^a^	6.91 ± 0.16 ^ax^	6.73 ± 0.17 ^ax^	6.66 ± 0.12 ^a^	5.38 ± 0.42 ^b^	***
T2	6.77 ± 0.10 ^a^	6.72 ± 0.10 ^ay^	6.64 ± 0.14 ^abx^	6.55 ± 0.10 ^ab^	6.30 ± 0.12 ^b^	***
Sig.	ns	**	**	ns	ns	

ND = Not determined. ^a–e^ Mean values in the same row not followed by a common letter differ significantly among storage times. ^x–y^ Mean values in the same column not followed by a common letter differ significantly among treatments. Sig. Significance; ns: not significant; * *p* < 0.05; ** *p* < 0.01; *** *p* < 0.001.
